# Prognostic value of liver stiffness measurement for the liver-related surgical outcomes of patients under hepatic resection: A meta-analysis

**DOI:** 10.1371/journal.pone.0190512

**Published:** 2018-01-11

**Authors:** Zitong Huang, Jingjing Huang, Tianran Zhou, Hongying Cao, Bo Tan

**Affiliations:** 1 School of Fundamental Medical Science, Guangzhou University of Chinese Medicine, Guangzhou, China; 2 The Research Center for Basic Integrative Medicine, Guangzhou University of Chinese Medicine, Guangzhou, China; 3 School of Chinese Materia Medica, Guangzhou University of Medicine, Guangzhou, China; Yonsei University College of Medicine, REPUBLIC OF KOREA

## Abstract

**Background:**

Previous studies have discussed the liver stiffness measurement (LSM) performance on predicting liver-related surgical outcomes for patients of hepatocellular carcinoma (HCC) under hepatic resection, yet there is much variation in reporting and consistency of findings. Therefore, we report a meta-analysis on this issue.

**Methods:**

We comprehensively searched PubMed, Embase, and Web of science to find the eligible cohort studies. The pooled Odds Ratios (OR) and 95% confidence intervals (CIs) were calculated to evaluate effect. The weighted mean LSM value was calculated as the optimal LSM cut-off value among studies.

**Results:**

12 prospective cohort studies and one retrospective cohort study, including a total of 1942 cases were identified. The pooled results showed that preoperative LSM is significantly associated with the occurrence of overall postoperative complications (OR 1.76, 95% CI 1.46–2.11). In addition, a weighted mean LSM value of 14.2 kPa and 11.3KPa were suggested as the optimal LSM cut-off value reference using transient elastoqraphy (TE) for predicting overall postoperative complications in Asia countries and European countries, respectively.

**Conclusions:**

Preoperative LSM should be taken into account cautiously in the management of patients undergoing hepatectomy of HCC. Future studies could focus on setting a prognostic model integrated with LSM in predicting post-hepatectomy outcomes.

## Introduction

Hepatic resection is widely accepted as the major curative therapy for hepatocellular carcinoma (HCC), especially in patients with well-compensated cirrhosis[1]. Although advances have been made in perioperative care and surgical techniques, postoperative complications remain the main cause of unsatisfactory outcomes after resection of HCC[2]. Previous studies have identified cirrhosis or liver fibrosis as a negative factor contributing to liver decompensation and mortality after hepatectomy[3]. Hepatic resection may induce postoperative complications due to insufficient liver remnant volume. Therefore, careful preoperative assessment of liver reserve function is important for patient selection or the extent of liver resection to ensure the surgery safety.

The main conventional methods for preoperative assessment of liver function are Child-Pugh score, the model for end-stage liver disease score, and the use of Indocyanine green retention rate at 15 min[4]. Liver biopsy and hepatic venous pressure gradient can provide a more accurate evaluation but are invasive procedures.

Magnetic resonance elastography (MRE) and ultrasound elastography, including transient elastography (TE), shear wave elastography (SWE) and acoustic radio force impulse (ARFI) imaging technology have been reported as a noninvasive and convenient test to measure liver stiffness and distinguish the degree of liver fibrosis and cirrhosis to reflect objectively liver functional reserve[5, 6]. Some prospective cohort studies have indicated that preoperative liver stiffness measurement (LSM) is a valid tool for the prediction of post-hepatectomy liver failure (PHLF), hepatic liver insufficiency, and ascites in patients undergoing hepatic resection for HCC. Recent several meta-analyses had investigated that LSM was used for the assessment of liver fibrosis and cirrhosis degree, as well as the risk factor for liver decompensation, HCC, and death in patients with chronic liver diseases [7, 8]. However, to the best of our knowledge, no other study has examined its utility in surgical judgment. Therefore, we performed this meta-analysis to clarify further prognostic value of LSM for liver-related outcomes of patients after hepatic resection.

## Methods

This meta-analysis was conducted following the guidance of Cochrane Handbook[9] and suggestions provided in the study by Singh S[10]. We also followed the Preferred Reporting Items for Systematic Reviews and Meta-Analyses guidelines[11].

### Literature search strategy

We performed a systematic literature search in electronic databases of PubMed (January 1, 1966 through September 1, 2017), Embase (January 1, 1988 through September 1, 2017), and Web of science (January 1, 1993 through September 1, 2017) for articles published. The following key words and their combinations were searched in [Title/Abstract]: “liver,” “hepatic,” “stiffness,” “elastography,” “Fibroscan,” “hepatocellular carcinoma,” “prognosis,” “predict,” “resection,” “hepatectomy,” “operative,” and “surgical treatment.” Then, the major conferences (The Liver Meeting, organized by the American Association for the Study of the Liver; The International Liver Congress, organized by the European Association for the Study of the Liver; Digestive Diseases Week, organized in conjunction with the American Gastroenterological Association; and Congress of the International Hepato- Pancreato-Biliary Association) were searched manually for studies published in abstracts. In addition, the related-articles function was used to identify any potential studies missed by the electronic search. When the same population was described in two studies, the newly published or complete one was included. If necessary, we contacted the corresponding author to get further information. Two authors independently finished the literature search process (Zitong Huang and Jingjing Huang).

### Selection criteria

All available prospective or retrospective cohort studies that met the following criteria were included: (1) LSM was performed preoperatively in patients undergoing hepatic resection; (2) correlation of LSM with liver-related surgical outcomes (postoperative complications including PHLF, ascites and hepatic insufficiency) were described; and (3) the studies reported a measure of association—odds ratio (OR), or the sufficient data for their calculation were provided. The included studies were not restricted by language and study size. Specific types of literature such as letters to the editor and review articles comments were excluded.

### Quality assessment of studies

The quality assessment was independently preformed by two authors (Zitong Huang and Jingjing Huang), using the Newcastle–Ottawa Scale (NOS). Disagreements were resolved by consensus or by the third investigator (Tianran Zhou). This scale mainly comprises three quality parameters: patient selection, study comparability, and outcome assessment. Studies with NOS scores of ≥7 were regarded as high quality.

### Data extraction

All data were abstracted by one investigator (Zitong Huang) and verified by another independent investigator (Jingjing Huang). Conflicts were resolved by consensus or by the third investigator (Tianran Zhou). Information taken from each study included primary author, year of publication, country, patient age and sex, study follow-up period, underlying liver diseases, stage of fibrosis, Child-Pugh score, development of liver-surgical outcomes and its definition, the corresponding OR with 95%CI, body mass index, technique of LSM, range of LSM value, cut-off value and its sensitivity and specificity of LSM for predicting outcomes, and methods of data analyses. Study authors were contacted to obtain pertinent data.

### Definition

The primary end point was postoperative liver-related complications categorized by the modified Clavien–Dindo classification[12], including PHLF (defined according to the International Study Group of Liver Surgery definition or “50–50” criteria), hepatic ascites and insufficiency. Hepatic insufficiency was defined as persistent hyperbilirubinemia (total bilirubin level>5 mg/dl) for more than 5 days after surgery or postoperative death without other causes[13].

### Statistical analysis

The pooled OR with its 95% CI was calculated to estimate the association of LSM and surgical treatment outcomes. The predictive ability of LSM was assessed by the receiver operating characteristic (ROC) curve and corresponding area under the ROC (AUROC) curve. To evaluate the optimal cut-off value of LSM, we used the method performed in the study by Chon YE[7]. Briefly, the cut-off value and sample size of individual study were calculated to make a weighted mean value as the optimal cut-off value of these studies. Heterogeneity among the studies was assessed by the Cochran’s *Q* statistic and *I*^2^ tests. Either *P*<0.10 or *I*^2^ statistic >50% defined significant heterogeneity among the studies. In this case, the random effects model was performed; otherwise, the fixed effects model was implemented. In order to explore potential sources of heterogeneity, subgroup analysis and meta-regression were conducted. We further performed sensitivity analysis by omitting some individual studies or low-quality studies. Stata software version 12.0 (College Station, TX, USA) was used to perform in the meta-analysis.

## Results

### Study selection

**Fig 1:**
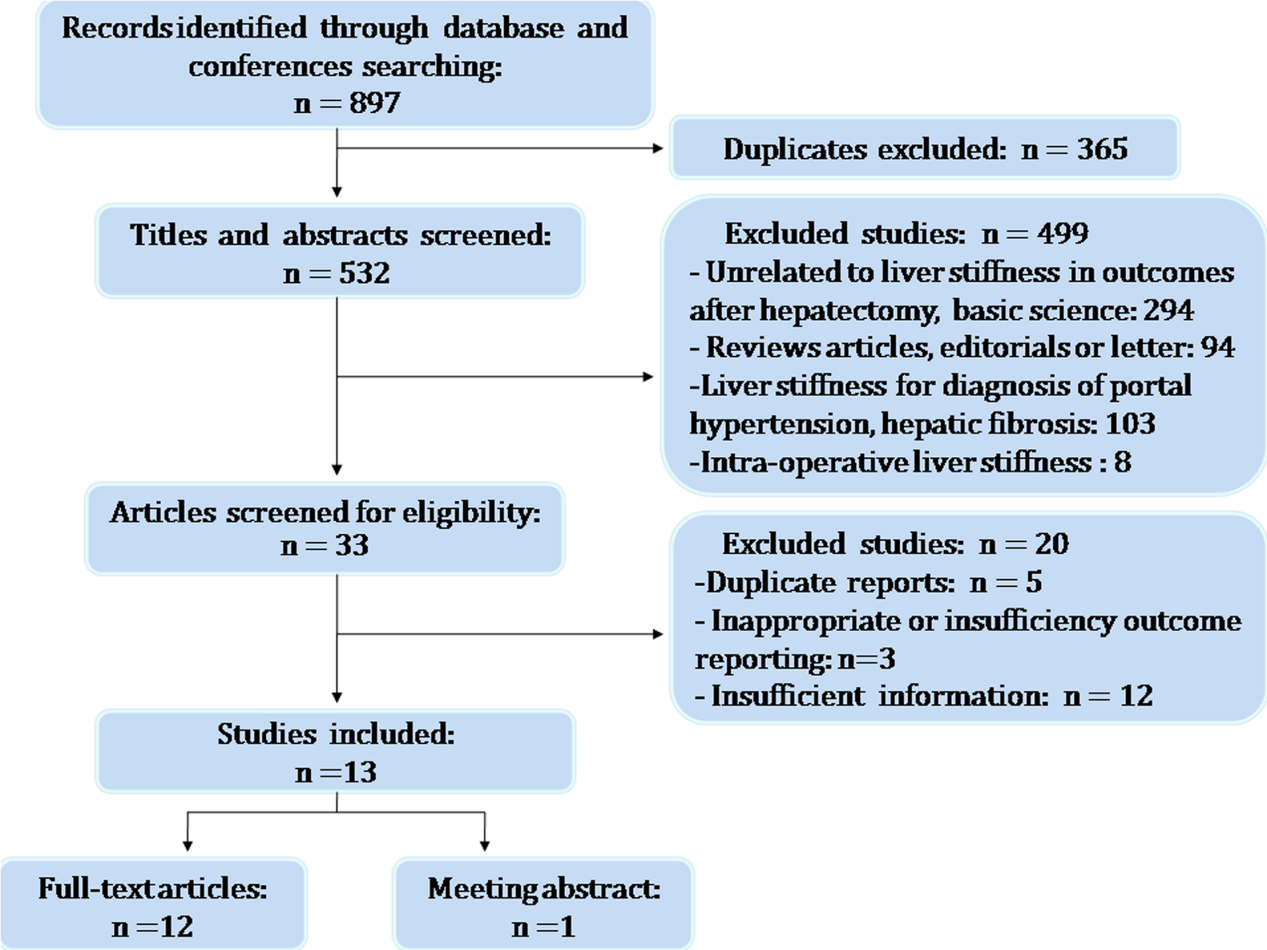
Flow diagram of the study selection process.

The initial search found a total of 897 articles. Following the exclusion of duplicate publications, 532 records were left. After screening the titles and abstracts, another 499 articles were excluded due to the following reasons: unrelated to liver stiffness in surgical outcome after hepatectomy, basis science(294), reviews articles, editorials or letter(94), LSM for diagnosis of portal hypertension(67) and hepatic fibrosis(36), intra-operative LSM(8). Another 20 articles were eliminated as the reporting outcomes were inappropriate or insufficiency and the data were insufficient or duplicated. Finally, 13 eligible studies were included in the present meta-analysis (Fig 1). The coefficient of agreement between the 2 investigators for study selection (Cohen’s *K* = 0.89; 95% CI, 0.78–0.99) was very good.

### Characteristics of the included studies

Among these 13 cohort studies, 10 were in Asia and 3 were in a European country, Italy. 7 studies used TE for measurement of liver stiffness; two used SWE, MRE, and ARFI imaging technology, respectively. The LSM value in patients ranged from 2.5 kPa to 75 kPa. Patients with Child-Pugh C liver function were excluded from the study. The majority of patients included were Child–Pugh class A. All studies, except one, used the receiver operating characteristic (ROC) curve and corresponding area under the ROC (AUROC) curve to assess the predictive ability of LSM; one studies used decision curve analysis. The surgical end point of the 13 studies was postoperative complications. Among these studies, PHLF was specially reported in seven studies, postoperative complications in three studies, ascites in two studies, and hepatic insufficiency in one study. Most of included patients had viral hepatitis, and the others had other chronic liver diseases, including non-alcoholic steatohepatitis and alcohol liver disease. Two Asian studies were performed exclusively in patients with viral hepatitis B. All of the studies included a mix of patient population at varying stages of fibrosis or cirrhosis (defined based on liver biopsy or LSM). The main characteristics of the included studies are summarized in Tables 1 and 2. The coefficient of agreement between two investigators for data extraction (Cohen’s *K* = 0.86; 95% CI, 0.75–0.96) was excellent.

**Table 1:** The fundamental features of the included studies.

Study	Country	Case number	Mean age (years)	Gender (M/F)	Time period	Etiology	Outcomes	LSM techniques	NOS Value
Shen 2017[14]	China	280	56.1	240/40	2015–2016	HBV, 100%	PHLF	SWE	6
Han 2017[15]	China	77	59	63/14	2014–2015	HBV,89.6% Others,10.4%	PHLF	SWE	7
Chong 2017[16]	China	255	58.6	218/37	2010–2014	HBV,81.6% HCV,6.7% Others,11.7%	PHLF	TE	7
Abe 2017[17]	Japan	175	69	123/52	2014–2016	HBC,19.4% HCV,23.6% Others,57.1%	complications	MRE	8
Nishio 2016[18]	Japan	177	68	140/37	2011–2014	HBC,18.6% HCV,37.3% NASH,16.3% EtOH,13.0% Others,14.7%	PHLF	ARFI	7
Lee 2016 [19]	China	144	58.9	106/38	2010–2013	HBV,80.5% HCV,11.1% EtOH,3.5% NBNC,3.5% Others,1.4%	PHLF	MRE	8
Donadon 2016[20]	Italy	240	65	225/115	2012–2015	HBV,2% HCV,14% EtOH,9% Others,75%	complications	TE	6
Cucchetti 2016[21]	Italy	202	64	171/31	2008–2014	HBV,17.8% HCV,63.9% Others,18.3%	PHLF	TE	8
Li 2015[22]	China	75	52.15	59/16	2012–2014	HBV,100%	PHLF ascites	TE	7
Wong 2013[23]	China	105	59	82/23	2010–2011	HBV,66.7% HCV,4.8% EtOH,1.9% NBNC,5.7% Others,21%	complications	TE	8
Harada 2012[24]	Japan	50	68	36/14	2009–2010	HBV,10% HCV,68% Others,22%	ascites	ARFI	8
Cescon 2012[25]	Italy	90	64	77/13	2008–2011	HBV,17.8% HCV,65.6% Others,16.6%	PHLF	TE	8
Kim 2008[26]	Korea	72	54.9	56/16	2006–2007	HBV,83.3% HCV,12.5% Others,4.2%	hepatic insufficiency	TE	7

M/F, Male/female; LSM, liver stiffness measurement; PHLF, Post-hepatectomy Liver Failure; HBV, hepatitis B-virus; HCV, hepatitis C-virus; NOS, Newcastle–Ottawa Scale; TE, transient elastography; SWE, shear wave elastography; MRE, magnetic resonance elastography; ARFI, acoustic radio force impulse; ISGLS, International Study Group of Liver Surgery definition; EtOH, alcohol liver disease; NASH, non-alcoholic steatohepatitis; NBNC, non-hepatitis B and non-hepatitis C; NA, data not available.

**Table 2:** Diagnostic data of each studies evaluating the performance of TE for postoperative complications.

Study	LSM cut-off value(KPa)	Sensitivity (%)	Specificity (%)	BMI(Mean)	AUROC
Chong 2017	12	83	73	23.7	0.83
Donadon 2016	9.7	88.9	67.3	25.1	0.728
Li 2015	14.3	100	76.1	NA	0.915
Wong 2013	12	85.7	71.8	23.2	0.79
Cescon 2012	15.7	96.1	68.7	24.8	0.865
Kim 2008	25.6	71.4	88.6	24.0	0.824

LSM, liver stiffness measurement; PHLF, Post-hepatectomy liver failure; BMI, body mass index; AUROC, area under the receiver operating characteristic curve; KPa, Kilopascal; NA, data not available.

### Overall postoperative complications after hepatectomy

**Fig 2:**
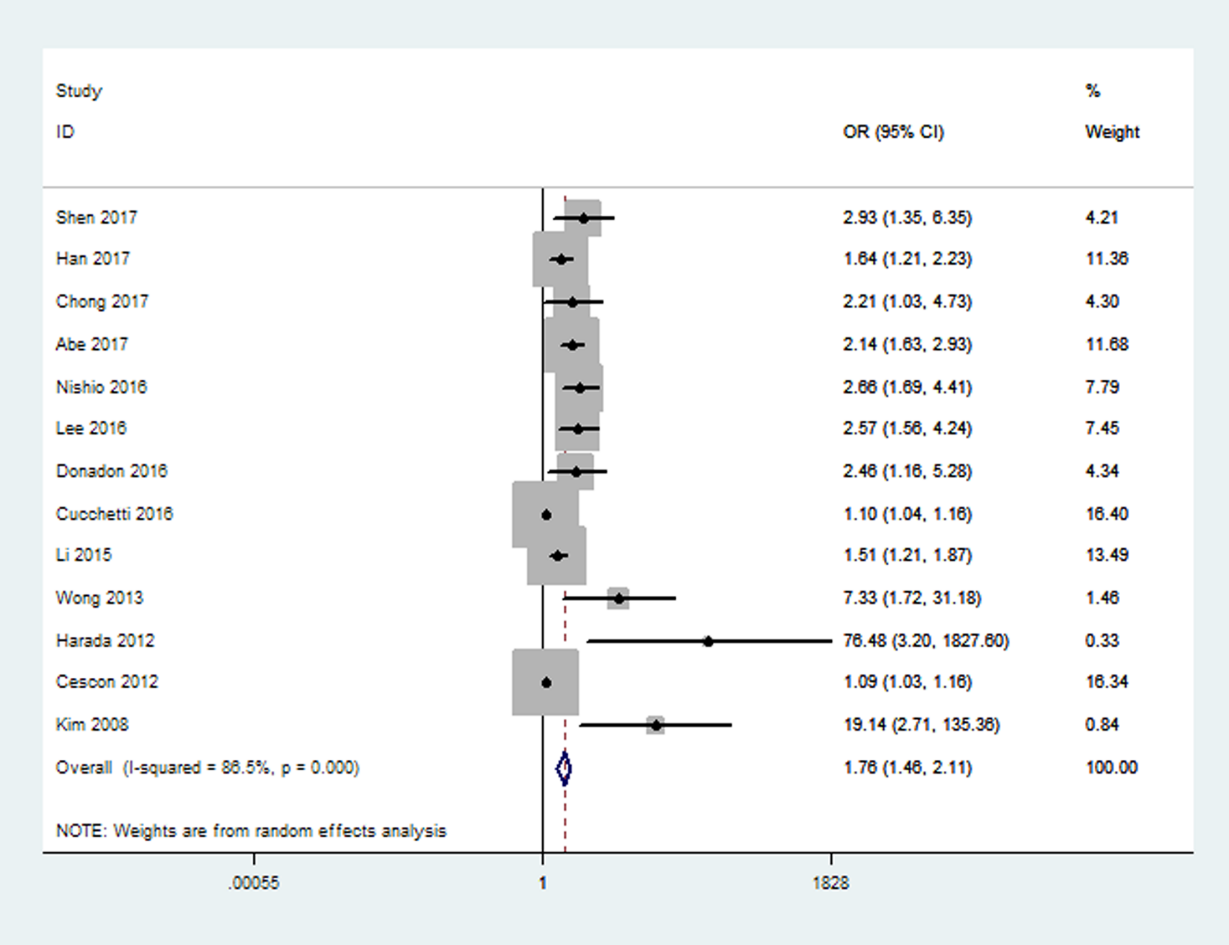
Forest plot on the associations between LSM and overall postoperative complications.

A total of 1942patients were included and analyzed for the prognostic significance of LSM for liver-related surgical outcomes. The relationship LSM and overall postoperative complications after hepatectomy was evaluated in 13 studies. The pooled results of the meta-analysis revealed that preoperative LSM was significantly associated with the development of overall postoperative complications (OR 1.76, 95% CI 1.46–2.11, Fig 2).

### Optimal cut-off value of LSM using TE for overall postoperative complications

**Fig 3:**
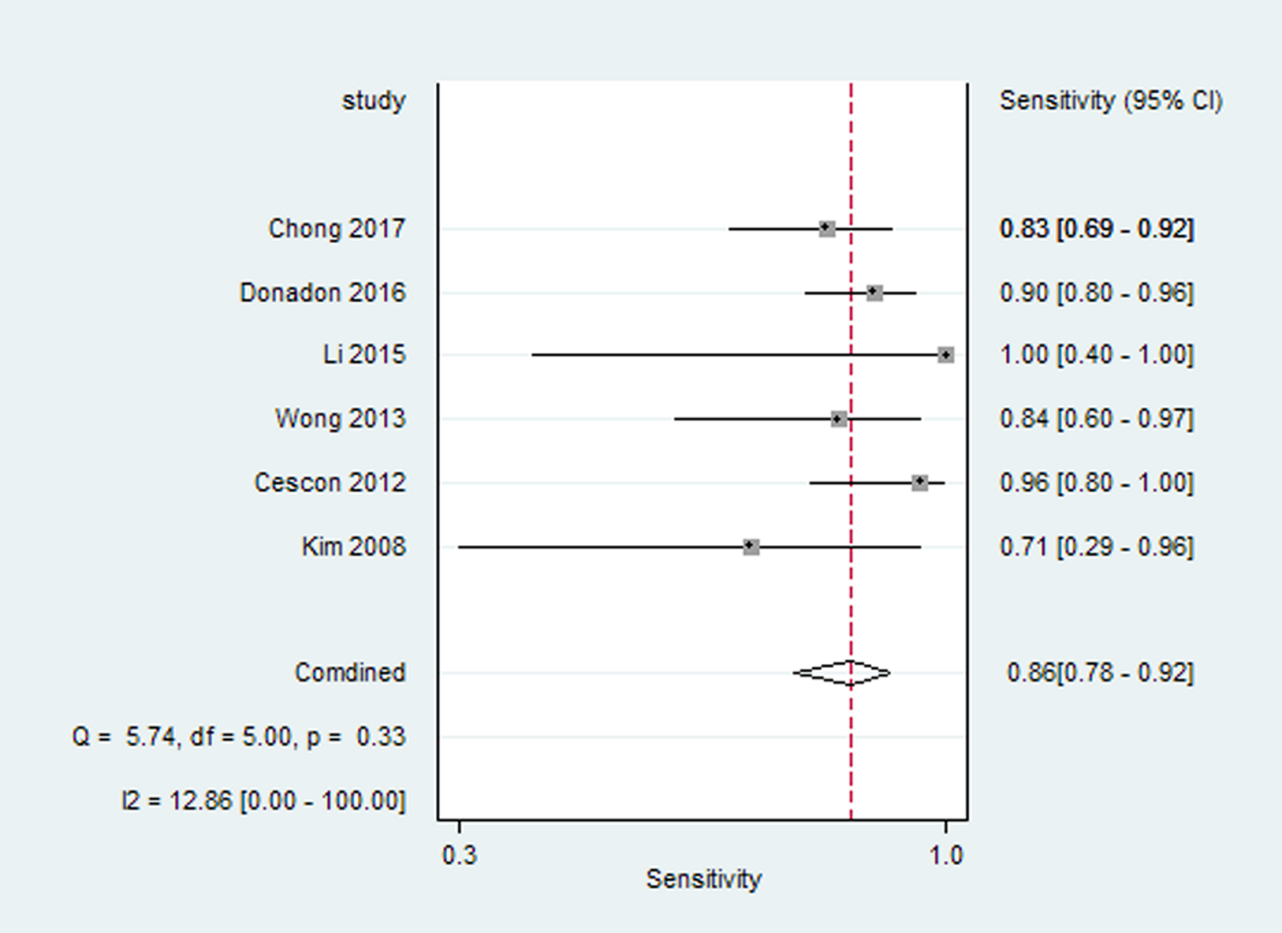
Forest plot meta-analyses of studies evaluating the sensitivity of live stiffness measured by TE to predict the overall postoperative complications.

**Fig 4:**
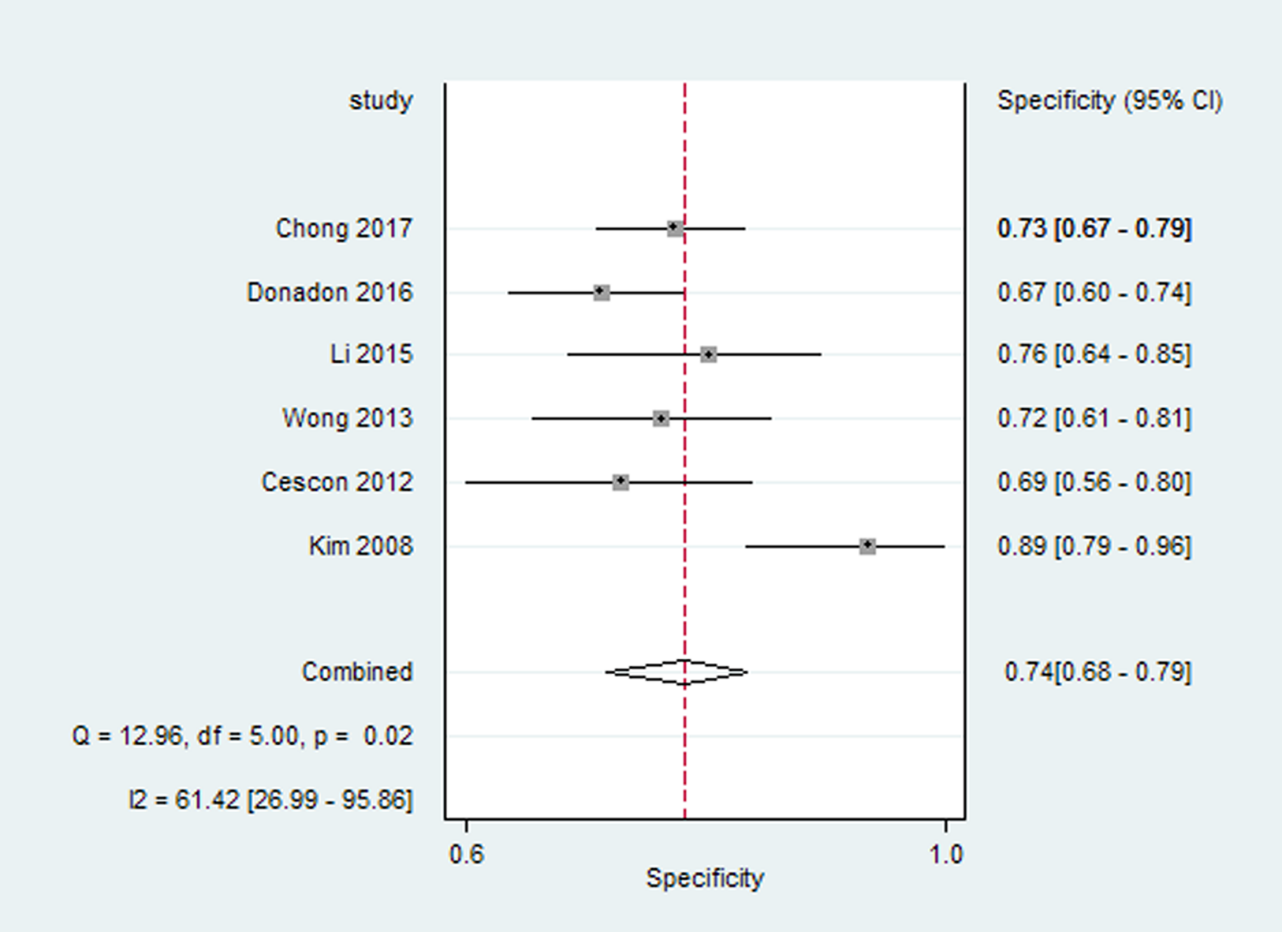
Forest plot meta-analyses of studies evaluating the specificity of live stiffness measured by TE to predict the overall postoperative complications.

**Fig 5:**
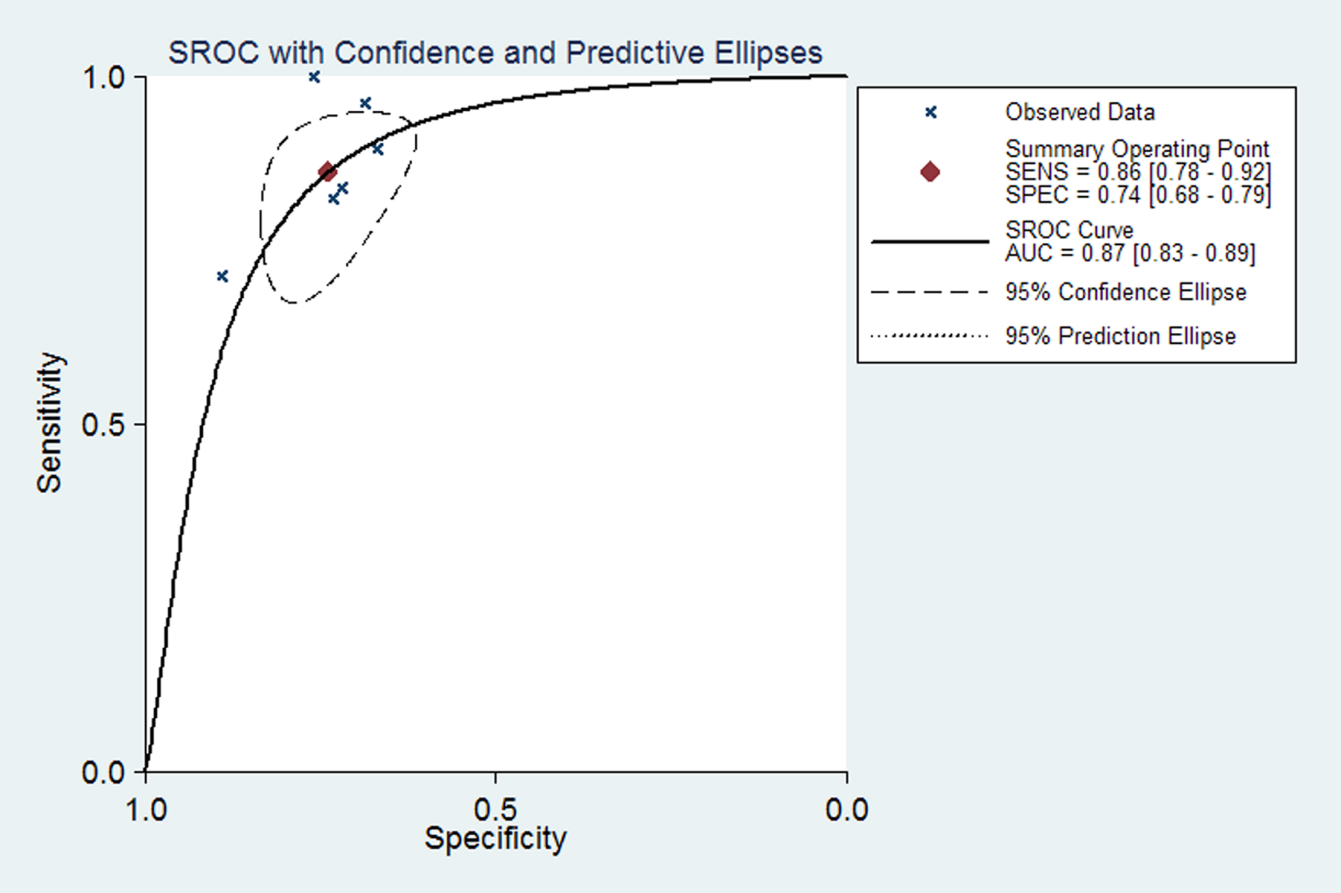
SROC curves for 6 studies of live stiffness measured by TE to predict the overall postoperative complications.

Six studies reported the preoperative LSM cut-off value using TE and diagnostic accuracy parameters (sensitivity, specificity and AUROC) for overall postoperative complications after hepatectomy. For the diagnosis of postoperative complications by TE, the pooled estimate for sensitivity was 86% (95% CI 78%, 92%) (Fig 3), and the pooled estimate for specificity was 74% (95% CI 68%, 79%) (Fig 4). The AUC of the live stiffness measured by TE to predict the overall postoperative complications was 0.87(95% CI 0.83, 0.89) (Fig 5). The LSM cut-off value using TE for postoperative complications varied from 9.7kPa to 25.6kPa. To ascertain the optimal LSM cut-off value, we made a weighted mean LSM value of these studies. The results showed that the weighted mean value of 14.2kPa and 11.3kPa as the optimal LSM cut-off value using TE for predicting postoperative complications in Asian and European countries, respectively (Table 3).

**Table 3:** Results of optimal LSM cut-off value using TE in predicting postoperative complications.

Country	Studies	Patients(n)	Weighted Mean LSM value(KPa)	Range(KPa)
Asia countries	4	507	14.2	12–25.6
European countries	2	330	11.3	9.7–15.7
overall	6	837	13.1	9.7–25.6

LSM, liver stiffness measurement; TE, transient elastography; KPa, Kilopascal.

### Subgroup analyses and meta-regression analyses

Considerable heterogeneity was observed in the overall analysis (*I*^2^ = 86.5%, *P* = 0.000) and the random effects model was adopted. There was significant inconsistency in the magnitude of effects and not in the direction of effect. Subgroup analyses and multivariable meta-regression were performed to explore sources of heterogeneity. Subgroup analysis (Table 4) indicated that the technique of LSM used, etiology of CLD and overall postoperative complications might result in the clinical heterogeneity. Further meta-regression analysis suggested that location of countries might be a potential source of heterogeneity (*P* = 0.033). Subgroup analysis and meta-regression indicated that percentage of patients with advanced fibrosis could not explain the heterogeneity. Summarized ORs of 2 studies using MRE (OR 2.24, 95% CI 1.74–2.89) and 2 studies using SWE (OR 1.98, 95% CI 1.60–3.30) was higher as compared with seven studies using TE (OR 1.30, 95% CI 1.11–1.52). The pooled ORs of ARFI subgroup demonstrated that LSM using ARFI had no significant effect on overall complications (OR 9.74, 95% CI 0.39–240.25). Additionally, the pooled ORs of ascites subgroup were 7.70 (95% CI 0.17–341.88), which had no significant association with LSM.

**Table 4:** Subgroup analyses for association between LSM and overall postoperative complications.

Group factors	Subgroup	studies	Combined OR	95%CI	Heterogeneity	*P* value
					I^2^	
Regions	Asia	10	2.30	1.75, 3.04	63.2%	0.004
	Europe	3	1.12	1.00, 1.26	78.5%	0.010
Technique of LSM	MRE	2	2.24	1.74, 2.89	0.0%	0.536
	TE	7	1.30	1.11,1.52	48.3%	0.085
	SWE	2	1.98	1.16, 3.30	46.1%	0.173
	ARFI	2	9.74	0.39, 240.25	76.2%	0.040
Outcome	Composite complications	3	2.87	1.65, 5.00	47.3%	0.150
	PHLF	7	1.46	1.23, 1.74	84.7%	0.000
	ascites	2	7.70	0.17, 341.88	82.9%	0.000
	Hepatic insufficiency	1	19.14	2.71, 135.27	NA	NA
Etiology of CLD	Mixed	11	1.74	1.42, 2.13	87.0%	0.000
	Viral hepatitis	2	1.89	1.02, 3.49	62.0%	0.105
Stage of fibrosis	The percentage of patients with fibrosis grade F4≥50%	7	1.32	1.11, 1.57	80.4%	0.000
	The percentage of patients with fibrosis grade F4<50%	6	2.20	1.60, 3.01	64.2%	0.016

LSM, liver stiffness measurement; TE, transient elastography; SWE, shear wave elastography; MRE, magnetic resonance elastography; ARFI, acoustic radio force impulse; CLD, chronic liver diseases.

### Sensitivity analysis

All studies were sequentially removed to evaluate the effect of individual study on the pooled ORs (Table 5). The pooled ORs of sensitivity analyses varied from 1.67(95%CI, 1.39–2.00) to 2.20(95%CI, 1.62–3.00) for the prognostic value of liver stiffness measurement for overall postoperative complications, suggesting that the pooled ORs were not significantly affected by individual study.

**Table 5:** Effect of individual studies on the pooled ORs of the prognostic value of liver stiffness measurement for overall postoperative complications.

Study omitted	OR	LCI	HCI
Shen 2017	1.71	1.42	2.05
Han 2017	1.77	1.46	2.14
Chong 2017	1.73	1.44	2.09
Abe 2017	1.67	1.39	2.00
Nishio 2016	1.67	1.39	2.00
Lee 2016	1.68	1.40	2.02
Donadon 2016	1.72	1.43	2.07
Cucchetti 2016	2.20	1.62	3.00
Li 2015	1.80	1.48	2.20
Wong 2013	1.71	1.42	2.04
Harada 2012	1.72	1.44	2.06
Cescon 2012	2.20	1.62	3.00
Kim 2008	1.70	1.43	2.04

## Discussion

This meta-analysis of 13 cohort studies on 1942 patients showed that LSM can significantly predict the occurrence of overall postoperative complications, which include PHLF, hepatic insufficiency, and ascites for patients with HCC undergoing hepatic resection. The result showed that preoperative LSM was significantly higher in patients who developed postoperative complications than in those who did not. Regardless of the varied baseline of LSM, a positive correlation was observed with the development of different grades of postoperative complications. Patients with a higher LSM will have a higher chance of getting high-grade complications.

Prediction on patients with high risk of poor surgical outcomes before hepatectomy allows surgeons to select appropriate candidates and improve chances for cure. Liver stiffness is closely related to liver function and reflects the possible volume of remnant liver after hepatectomy, which determine the risk of postoperative complications[27]. Additionally, Llop E et al. reported that TE can predict the occurrence of portal hypertension (hepatic venous pressure gradient≥ 10 mm Hg) in about 50% of patients, particularly in patients with LSM>21kPa[28]. Portal hypertension has been proven as a risk factor for postoperative complications[29]. Accordingly, the LSM prognostic value can also account for the presence of portal hypertension in some patients.

The optimal LSM cut-off value varied between the included studies. Firstly, this variation can be explained by the different definitions of postoperative complications in studies that range from hepatic insufficiency to fulminant hepatic failure with high mortality. In the study by Hong Han et al., the LSM cut-off value at 6.9 kPa was used to identify PHLF grade A/B; whereas in the study by Seung Up Kim et al., the outcomes were hepatic insufficiency, which was defined as persistent hyperbilirubinemia for more than 5 days after surgery or postoperative death without other identifiable cause. As a result, a high LSM cut-off value at 25.7kPa was detected because it caused a high risk of irreversible PHLF. Secondly, LSM varied in patient groups with different backgrounds, such as nonalcoholic fatty liver disease and viral hepatitis[30]. Thirdly, in comparison with the European population, the body mass index was smaller in the Asian cohort. A higher prevalence of hepatitis C infection was also observed in European countries, whereas hepatitis B infection in Asian countries. If the major cause of liver diseases was the hepatitis B virus infection, the liver tends to become macronodular and the total amount of fibrotic material maybe lower than that in the hepatitis C virus infection or other causes[31]. Moreover, Wong et al. and Chong et al. recommended LSM >12kPa as a predictor for the development of major complications after hepatectomy for various pathologies. In this meta-analysis, we calculated weighted mean LSM value of TE separately in European and Asian countries. The cut-off value can be >14.2kPa in Asian patients and >11.3kPa in European patients of HCC, in reference to predict overall postoperative complications.

Predicting the high risk postoperative complications before surgery is significant because its prevention is more important than treatment. Appropriate choice of preoperative technique to reduce the risk of complications and excellent management for high risk patients are essential to optimize surgical outcomes. The cutoff value of LSM at 14.2kPa in Asian patients and 11.3kPa in European patients can be used to stratify patients who need extra peri-operative care. Before hepatectomy, techniques like portal vein embolization should be considered to improve the functional liver remnant and minimize intra-operative hepatocyte injury that would be caused by the abrupt increase in portal venous pressure at the time of resection[32]. In order to preserve more liver parenchyma, precise hepatectomy should be performed. At the time of surgery, factors associated with increased risk should be avoid and included massive operative blood loss, blood transfusions, vascular resection and extended operative time[33]. The time of portal occlusion must be strictly assessed and half hepatic blood flow occlusion is recommended. After operation, close postoperative monitoring and early supportive treatment to save the function of remnant liver are critical for survivals. Supporting care of failing systems, infusion of albumin, blood transfusion and nutritional supplementation are the main treatments of postoperative complications[34].

Significant heterogeneities were found among studies when we investigated the associations between LSM with composite postoperative complications, which included PHLF, hepatic insufficiency, and ascites. The random-effects model was used in the pooling of data to achieve relatively narrow CIs. This method might reduce the effect of heterogeneity but not abolish it. The heterogeneities were seen mainly in the strength of the association between LSM and surgical outcomes but not in the direction of association. To explore the sources of heterogeneities, both the subgroup analyses and meta-regression were performed. The result showed that technique of LSM used, location of countries, and overall postoperative complications might contribute to the heterogeneities. TE, SWE, and ARFI imaging technology are three noninvasive methods of ultrasound elastography. TE and SWE are all shear wave-based elastography, which uses a mechanical vibrator to apply shear stress to the target tissue[35]. Numerous investigations have extensively validated that these elastography methods had similar diagnostic performances for predicting cirrhosis and liver fibrosis in patients with CLD[36]. Moreover, liver stiffness can be measured with MRE in patients who are obese or have ascites, whereas the shear waves used with ultrasound elastography are limited for these problems[37, 38]. In this study, the results showed that these elastography techniques have similar positive effect as predictors for postoperative complications.

Several potential limitations must be taken into account in this study. The study by Altman DG had shown the intrinsic limitations of meta-analysis of prognostic studies, mainly owing to the quality of the primary studies and the possibility of publication bias on non-significant reports[39]. Clinical heterogeneity of patients and poor assessing methodological quality of prognostic studies have been found, as well as influence in treatment on follow-up evaluation. In this regard, several heterogeneities remain unexplained. The limited number of the included studies is also a limiting factor. Thus, insufficient data to perform all the preplanned subgroup analyses was found. Statistical tests were not performed for funnel plot asymmetry because of the number of studies and the considerable heterogeneity.

In summary, these data converged into a conclusion that LSM is a useful preoperative predictor of the development of postoperative complications in patients with HCC undergoing hepatectomy. Future studies can focus on setting a prognostic model integrated with LSM in predicting post-hepatectomy outcomes.

## Supporting information

S1 Table Completed 2009 PRISMA checklist. (DOC)

S2 Table The raw data extracted from studies. (DOC)
