# Supplementary material for: Prognostic value of liver stiffness measurement for the liver-related surgical outcomes of patients under hepatic resection: A meta-analysis
Source: PLoS One. 2018 Jan 11;13(1):e0190512. doi: 10.1371/journal.pone.0190512 (PMC5764309; doi:10.1371/journal.pone.0190512)
Supplement: S2 Table — (DOC) [file pone.0190512.s002.doc]

1. Summary table

| Author | Year | Country | Study type | Reasons for liver resection | n | Male | Female | Mean age  (year) | Time period  (year) | Etiology | Outcomes  (definition) | LSM  techniques | Data analyses | Quality  Value |
| --- | --- | --- | --- | --- | --- | --- | --- | --- | --- | --- | --- | --- | --- | --- |
| Shen et al | 2017 | China | retrospective cohort | HCC | 280 | 240 | 40 | 56.1 | 2015-2016 | HBV, 100% | PHLF(ISGLS) | SWE | multivariate analyses | 6 |
| Han et al | 2017 | China | prospective cohort | HCC | 77 | 63 | 14 | 59 | 2014-2015 | HBV,89.6%  Others,10.4% | PHLF(ISGLS) | SWE | multivariate analyses | 7 |
| Chong et al | 2017 | China | prospective cohort | HCC | 255 | 218 | 37 | 58.6 | 2010-2014 | HBV,81.6%  HCV,6.7%  Others,11.7% | PHLF(ISGLS)  major complication | TE | multivariate analyses | 7 |
| Abe et al | 2017 | Japan | prospective cohort | HCC  liver metastasis | 175 | 123 | 52 | 69 | 2014-2016 | HBC,19.4%  HCV,23.6%  Others,57.1% | major complications(Dindo-Clavien classification) | MRE | multivariate analyses | 8 |
| Nishio et al | 2016 | Japan | prospective cohort | HCC | 177 | 140 | 37 | 68 | 2011-2014 | HBC,18.6%  HCV,37.3%  NASH,16.3%  EtOH,13.0%  Others,14.7% | PHLF(ISGLS) | ARFI | multivariate analyses | 7 |
| Lee et al | 2016 | China | prospective cohort | HCC | 144 | 106 | 38 | 58.9 | 2010-2013 | HBV,80.5%  HCV,11.1%  EtOH,3.5%  NBNC,3.5%  Others,1.4% | PHLF(ISGLS) | MRE | multivariate analyses | 8 |
| Donadon et al | 2016 | Italy | prospective cohort | HCC  liver metastases  other malignancies | 240 | 225 | 115 | 65 | 2012-2015 | HBV,2%  HCV,14%  EtOH,9%  Others,75% | complications | TE | multivariate analyses | 6 |
| Cucchetti et al | 2016 | Italy | prospective cohort | HCC | 202 | 171 | 31 | 64 | 2008-2014 | HBV,17.8%  HCV,63.9%  Others,18.3% | PHLF(ISGLS) | TE | multivariate analyses | 8 |
| Li et al | 2015 | China | prospective cohort | HCC | 75 | 59 | 16 | 52.15 | 2012-2014 | HBV,100% | PHLF(50-50criteria)  ascites | TE | multivariate analyses | 7 |
| Wong et al | 2013 | China | prospective cohort | HCC  liver metastasis | 105 | 82 | 23 | 59 | 2010-2011 | HBV,66.7%  HCV,4.8%  EtOH,1.9%  NBNC,5.7%  Others,21% | major complications  (Clavien classification) | TE | multivariate analyses | 8 |
| Harada et al | 2012 | Japan | prospective cohort | HCC | 50 | 36 | 14 | 68 | 2009-2010 | HBV,10%  HCV,68%  Others,22% | ascites | ARFI | multivariate analyses | 8 |
| Cescon et al | 2012 | Italy | prospective cohort | HCC | 90 | 77 | 13 | 64 | 2008-2011 | HBV,17.8%  HCV,65.6%  Others,16.6% | PHLF(Dindo-Clavien classification) | TE | multivariate analyses | 8 |
| Kim et al | 2008 | Korea | prospective cohort | HCC | 72 | 56 | 16 | 54.9 | 2006-2007 | HBV,83.3%  HCV,12.5%  Others,4.2% | hepatic insuficiency | TE | multivariate analyses | 7 |

**B. Diagnostic data**

| Author name | Year | Country | Zone | n | LSM cut-off value (KPa) | Range of LSM value (KPa) | Sensitivity (%) | Specificity (%) | AUROC |
| --- | --- | --- | --- | --- | --- | --- | --- | --- | --- |
| Chong et al | 2017 | China | Asia | 255 | 12 | 3.8-75 | 83 | 73 | 0.83 |
| Donadon et al | 2016 | Italy | Europe | 240 | 9.7 | 2.5-75 | 88.9 | 67.3 | 0.728 |
| Li et al | 2015 | China | Asia | 75 | 14.3 | NA | 100 | 76.1 | 0.915 |
| Wong et al | 2013 | China | Asia | 105 | 12 | NA | 85.7 | 71.8 | 0.79 |
| Cescon et al | 2012 | Italy | Europe | 90 | 15.7 | 5.3-58.2 | 96.1 | 68.7 | 0.865 |
| Kim et al | 2008 | Korea | Asia | 72 | 25.6 | NA | 71.4 | 88.6 | 0.824 |

**C. The association between LSM and overall postoperative complications**

| Author name | Year | Country | OR | L | U |
| --- | --- | --- | --- | --- | --- |
| Shen et al | 2017 | China | 2.929 | 1.351 | 6.351 |
| Han et al | 2017 | China | 1.642 | 1.208 | 2.231 |
| Chong et al | 2017 | China | 2.21 | 1.03 | 4.73 |
| Abe et al | 2017 | Japan | 2.14 | 1.63 | 2.93 |
| Nishio et al | 2016 | Japan | 2.66 | 1.69 | 4.41 |
| Lee et al | 2016 | China | 2.57 | 1.56 | 4.24 |
| Donadon et al | 2016 | Italy | 2.46 | 1.16 | 5.28 |
| Cucchetti et al | 2016 | Italy | 1.095 | 1.04 | 1.16 |
| Li et al | 2015 | China | 1.506 | 1.21 | 1.87 |
| Wong et al | 2013 | China | 7.33 | 1.72 | 31.18 |
| Harada et al | 2012 | Japan | 76.48 | 3.2 | 1,827.60 |
| Cescon et al | 2012 | Italy | 1.093 | 1.028 | 1.162 |
| Kim et al | 2008 | Korea | 19.14 | 2.71 | 135.36 |
